# Screening for comorbid autoimmune disease should be considered in children with ANA positive juvenile idiopathic arthritis – results from the south-Swedish juvenile idiopathic arthritis cohort

**DOI:** 10.1186/s12969-024-01030-x

**Published:** 2024-10-19

**Authors:** Alma Dahlberg, Helena Tydén, Anna Saxne Jöud, Fredrik Kahn, Elisabet Berthold

**Affiliations:** 1 Department of Clinical Sciences Lund, Pediatrics, Lund University, Lund, Sweden; 2 Skåne University Hospital, Lund and Malmö, Sweden; 3 Wallenberg Center for Molecular Medicine, Lund University, Lund, Sweden; 4 Department of Clinical Sciences Lund, Rheumatology, Lund University, Lund, Sweden; 5 Department of Laboratory Medicine, Division of Occupational and Environmental Medicine, Lund University, Lund, Sweden; 6 Department of Clinical Sciences Lund, Orthopedics, Lund University, Lund, Sweden; 7 Department of Research and Education, Skåne University Hospital, Lund, Sweden; 8 Department of Clinical Sciences Lund, Section of Infection Medicine, Lund University, Lund, Sweden

**Keywords:** Juvenile arthritis, Juvenile idiopathic arthritis, JIA, Comorbidity, Outcome, Autoimmunity, Hypothyroidism, Coeliac disease, Diabetes Mellitus Type 1

## Abstract

**Background:**

There is no consensus or clinical guidelines for screening routines of autoimmune disease in individuals with juvenile idiopathic arthritis (JIA), since results are conflicting whether the risk for such conditions is increased or not among individuals with JIA. The aim of this study was to investigate if the frequency of comorbid autoimmune conditions is increased after JIA diagnosis in a validated population-based JIA cohort in southern Sweden.

**Methods:**

Autoimmune comorbidities were evaluated in a pre-existing population-based JIA cohort of 302 participants, constituting of individuals diagnosed with a validated JIA diagnosis 2000–2010 in southern Sweden. The comorbidities were determined through analysis of diagnosis codes registered after the JIA diagnosis and until 2019. With the use of a reference population of 1510 age- and sex matched individuals, hazard ratios (HR) were calculated with Cox proportional models.

**Results:**

During the study period, 7.7% of the JIA cohort received an autoimmune diagnosis after their JIA diagnosis. Individuals with JIA had an increased risk of autoimmune diseases in general (HR 4.11, 95% CI 2.13–7.91) within the first 7 years of disease, as well as separately for coeliac disease (HR 5.24, 95% CI 1.76–15.65) and hypothyroidism (HR 3.74, 95% CI 1.14–12.30) compared to the reference population. Antinuclear antibody (ANA) positivity was associated with a significantly increased risk of comorbid autoimmune disease in the JIA cohort, with HR 6.21 (95% CI 1.64–23.55) for ANA positive individuals.

**Conclusions:**

Individuals with JIA have a significantly increased risk of being diagnosed with an autoimmune condition after receiving their JIA diagnosis compared to matched references. ANA positivity is associated with a further increased risk. Our results emphasize awareness in physicians of additional autoimmune disorders in individuals with JIA and advocate serological screening of autoimmune conditions during follow-up.

**Supplementary Information:**

The online version contains supplementary material available at 10.1186/s12969-024-01030-x.

## Background

Juvenile idiopathic arthritis (JIA) serves as an umbrella term for seven heterogenous arthritis subcategories, defined by joint inflammation with duration for at least six weeks, onset before the 16th birthday and unknown aetiology [1, 2]. The JIA subcategories are categorized by the number of inflamed joints, presence of extraarticular organ involvement, and serological features. The disease can hence present in various ways but share the feature of arthritis, possibly causing pain and mobility limitations leading to short- and long-term consequences. The present definition and classification criteria were proposed by the International League of Associations for Rheumatology (ILAR) and have been the international diagnostic standard since 2001 [2]. Due to heterogenous presentation in the same JIA subgroup, and since the current JIA criteria separate children from their adult counterparts by its specific paediatric arthritis definition, new subgroups have been proposed. A unique childhood specific subgroup without a clear adult equivalent is suggested, defined by presence of antinuclear antibodies (ANA) in early onset arthritis (diagnosis before the age of six), independent of the number of affected joints [3, 4].

JIA is the most common paediatric rheumatic disease with a reported incidence rate estimated to 12.8/100 000 children/year in southern Sweden [5]. Both genetic and environmental factors are believed to be involved in the pathogenesis [1]. Current treatment guidelines include non-steroid anti-inflammatory drugs, intra-articular corticosteroid injections and disease-modifying anti-rheumatic drugs (DMARD) [1]. Biological DMARDs (bDMARD) have been available as treatment for JIA in Sweden since 1999. JIA is a chronic disease with long-term follow-up data showing 45.6% of individuals diagnosed 1997–2000 having active disease after 18 years [6] and 41% of individuals diagnosed 1980–85 having active disease or being in remission on medication after 30 years [7]. Thus, a considerable number of individuals with JIA still have active disease persisting into adulthood. It is poorly established what impact JIA have on adult life in terms of risk of comorbidities due to autoimmunity, persistent inflammation, and/or immunomodulatory treatment.

Autoimmune disorders are more common conditions in Northern Europe as compared to other countries [8–10]. The estimated European prevalence of hypothyroidism in the adult population is 3–5% [10] and the Swedish prevalence of coeliac disease is approximately 2% in children [9]. Since genetic factors contribute to the development of autoimmune disease, autoimmune conditions tend to co-occur. Due to differences in study design and number of study participants, previous results on autoimmune comorbidity in JIA are conflicting and screening for autoimmune conditions in JIA is not uniformly recommended [11–14]. However, some studies implicate an elevated risk of comorbid autoimmune diseases in JIA [15–17]. Reports from Finland suggest a fivefold increased risk of type 1 diabetes mellitus, coeliac disease, and hypothyroidism in children with JIA compared to the general population [17], in contrast to Swedish results showing prevalence of coeliac disease among children with JIA similar to that in the general population, thus not recommending screening for coeliac disease in Swedish individuals with JIA [11]. However, screening for disease specific autoantibodies could detect organ-specific autoimmunity before the development of clinical autoimmune disease, potentially preventing morbidity and irreversible tissue damage. A recent multicentre registry study proposed the benefit of yearly serological screening for autoimmune thyroid disease in female ANA positive individuals with JIA and family history of autoimmune thyroid disease [18]. Therefore, before establishing uniform clinical guidelines regarding screening for autoimmune comorbidities in JIA with potential benefits of early diagnostic detection of a comorbid condition, more research is still needed.

The overall purpose of this study was to investigate whether persons with JIA have an increased risk of being diagnosed with a comorbid autoimmune condition after their JIA diagnosis in a validated population-based JIA cohort in southern Sweden, compared to a reference population matched for age, sex and residential region. The study further aimed to explore if the factors; sex, immunological data, and the need for DMARD treatment within the following calendar year after JIA diagnosis, could be used as predictors of comorbid autoimmune diagnosis.

## Methods

### Study area

For the study of autoimmune comorbidities in JIA we used a pre-existing south-Swedish JIA cohort with individuals collected in Skåne, the southernmost region of Sweden [5, 19]. In 2019, Skåne was the third largest region by population in Sweden with 1 377 827 inhabitants, constituting 13.3% of the Swedish population. Children aged 0–15 years accounted for 19.4% of the population [20]. The study area has one university hospital, nine other hospital-associated paediatric outpatient facilities, and six private paediatric outpatient facilities. The healthcare in Sweden is tax-funded, and the paediatric care is subsidized for all children. Additionally, children undergo regular controls both at child healthcare and in school. These factors, combined with mandatory diagnosis registration, diminishes the risk of missing to include persons in a population-based cohort, since symptoms suspicious of JIA rarely are neglected or missed at primary healthcare facilities.

### Study population

For this study, 302 individuals with a validated JIA diagnosis according to the 2001 ILAR criteria [2] with date of diagnosis between 1 January 2000 and 31 December 2010 were included from the previously published south-Swedish JIA cohort. The south-Swedish JIA cohort is a population-based cohort and consists of retrospectively collected JIA cases validated through medical record review. The case collection process for the south-Swedish JIA cohort is described in detail in previous publications [5, 19], but in short, was a two-step process. First, incident cases of juvenile arthritis 1980–2010 in the Swedish region Skåne were identified through a search for registered International Classification of Diseases (ICD)-codes of juvenile arthritis from both in- and outpatient care at the local database at the regional center for paediatric rheumatology, Lund, and at the diagnosis register at the National Board for Health and Welfare (NBHW). Secondly, to validate the diagnoses, medical records were reviewed, and the patients were subclassified according to the 2001 ILAR criteria [2]. Information about immunological data and annually prescribed pharmacological treatment was collected during the case collection process. Methotrexate, chloroquine phosphate, sulfasalazine, azathioprine, gold, mycophenolate, cyclosporine, penicillamine and chlorambucil was registered as conventional synthetic DMARDs (csDMARD), while tumor necrosis factor inhibitors, anakinra, tocilizumab and ustekinumab was registered as bDMARD.

JIA cases as well as references were followed until migration, death, diagnosis with an autoimmune disease, or end of study period 31 December 2019, whichever occurred first. Individuals with date of comorbid autoimmune disease before JIA diagnosis or within 90 days from JIA diagnosis were excluded, as were individuals lost to follow-up within the same period.

### Data source

We acquired registered diagnoses for autoimmune conditions between 1 January 1998 and 31 December 2019 from the regional administrative healthcare register, Skåne Healthcare Register (SHR). SHR includes information from healthcare visits in primary care, as well as from in- and outpatient hospital care visits in the region. The register was established in 1998 and contains diagnosis codes from public healthcare visits according to “International Statistical Classification of Diseases and Related Health Problems, tenth revision” (ICD-10). Due to reimbursement purposes, diagnosis code registration is mandatory in Skåne. Thus, SHR contains close to 100% assigned diagnosis codes per each healthcare visit from 1998 for inpatient care, and since 2004 also for outpatient care [21]. Data was acquired from SHR during 2020 after the study period ended, both for the JIA cohort and the references.

The acquired diagnosis codes analysed in this study were: hypothyroidism (E03), thyrotoxicosis (E05.0), autoimmune thyroiditis (E06.3), coeliac disease (K90.0), type 1 diabetes mellitus (E10), vitiligo (L80), and alopecia areata (L63). To minimize the risk of including persons with an incorrectly registered autoimmune diagnosis, a person was considered to have a verified autoimmune disease if they were diagnosed with any of the above-mentioned conditions at one inpatient healthcare visit or had at least two registered diagnosis codes at two separate outpatient healthcare visits (primary and specialized outpatient care) with a physician.

### Statistical Analyses

For the JIA cohort, a reference population with five individuals without JIA was collected from SHR, matched for year of birth, sex, residential region, and for having at least one healthcare visit during the study period (also used in previous studies [19]).

Demographics of the study population is presented with descriptive statistics. Conditional Cox proportional hazard regression models are used for calculation of hazard ratios (HR) with 95% confidence interval (CI) for all the autoimmune diseases, and for coeliac disease and hypothyroidism separately. These conditions were chosen for separate analysis since they are easily screened for with blood samples. Date of JIA diagnosis was used as inclusion variable. Also, we chose not to include the years 1998–1999 in the statical analyses to secure exclusion of prevalent comorbid autoimmune cases prior to 1998. We believe that the majority of the Swedish patients with autoimmune diseases have regular check-ups with at least two years interval. In total, 14 individuals with JIA were excluded due to having received an autoimmune diagnosis before or within 90 days from their JIA diagnosis, and 15 JIA cases were lost to follow-up before their JIA diagnosis. Among the reference population, 219 persons were excluded due to following causes; exclusion of their corresponding individual with JIA (*n* = 145), autoimmune diagnosis prior to study inclusion (*n* = 26), migration from the study area prior to study inclusion (*n* = 44), death prior to study inclusion (*n* = 2), and no available lost to follow-up date (*n* = 2). To investigate the impact of time from JIA diagnosis on the risk of comorbid diagnosis, the proportionality was investigated with Kaplan–Meier analysis, as were the residuals of the conditional Cox proportional hazard regression models.

Statistical calculations were made using readxl, dplyr, lubridate, survival, survminer in R 3.6.2 software (R Foundation for Statistical Computing; https://www.r-project.org/), and Statistical Package for the Social Sciences (SPSS), version 27 (IBM Corp., Armonk, N.Y.).

The study was conducted in accordance with the Declaration of Helsinki and approved by the Regional Ethical Board for southern Sweden (2011/379, 2013/192 and 2015/62) and the National Ethical Review Agency (2020–02935).

## Results

### Demographic information

Among the 302 persons diagnosed with JIA between 2000–2010, persistent oligoarthritis was the most common subgroup (36.8%), followed by undifferentiated arthritis (14.6%) and rheumatoid factor (RF) negative polyarthritis (12.6%) (Table 1). Approximately half the cohort was ANA positive, and 29.6% was categorized into the proposed JIA subgroup of early onset arthritis with ANA positivity. Two thirds of the cohort were female and the median age at diagnosis was 9.4 years. In the JIA cohort, 58.9% had ever been treated with a DMARD. A csDMARD had been prescribed for 58.6% of the total JIA cohort and 22.5% were at some point during their follow-up treated with a bDMARD.

**Table 1:** Demographic information

Characteristics	JIA cohort (*n* = 302)	References (*n* = 1510)
Female, n (%)	204 (67.5%)	1020 (67.5%)
Age at JIA diagnosis or cohort entry in years, median (IQR)	9.4 (3.5–13.1)	9.4 (3.5–13.1)
ANA positive, n (%)	150 (51.0%) *n* = 294	NA
ANA positive early onset, n (%)	88 (29.6%) *n* = 297	NA
ILAR category, n (%)		
* Systemic arthritis*	11 (3.6%)	NA
* Oligoarthritis*	145 (48.0%)	NA
* Polyarthritis, RF-*	38 (12.6%)	NA
* Polyarthritis, RF* +	17 (5.6%)	NA
* Psoriatic arthritis*	23 (7.6%)	NA
* Enthesitis-related arthritis*	24 (7.9%)	NA
* Undifferentiated arthritis*	44 (14.6%)	NA
DMARD history, n (%)		
* Any DMARD*	178 (58.9%)	NA
* csDMARD*	177 (58.6%)	NA
* bDMARD*	68 (22.5%)	NA

Clinical and serological characteristics of the included 302 individuals with juvenile idiopathic arthritis (*JIA*) and 1510 references from the south-Swedish *JIA* cohort, diagnosed 2000–2010

*Abbreviations: ANA* antinuclear antibodies, *DMARD* disease-modifying antirheumatic drug, *bDMARD* biological DMARD, *csDMARD* conventional synthetic DMARD, *ILAR* International League of Associations for Rheumatology, *IQR* Interquartile range, *NA* not applicable

### Autoimmune diagnosis was more common in the *JIA* group

During the study period excluding the wash-out period (- 2 years—+ 90 days after JIA diagnosis), 21 (7.7%) individuals with JIA were diagnosed with at least one of the autoimmune conditions; hypothyroidism, thyrotoxicosis, autoimmune thyroiditis, coeliac disease, type 1 diabetes mellitus, vitiligo, or alopecia areata after their JIA diagnosis (Table 2). During the same period 43 (3.3%) of the reference population was diagnosed with an autoimmune diagnosis. Hypothyroidism was the most common autoimmune condition in both the JIA cohort (3.3%) and reference population (1.5%). Coeliac disease was the second most common comorbidity in the JIA cohort (2.6%), while coeliac disease (0.7%) and type 1 diabetes mellitus (0.7%) were equally the second most common among the references. Only one individual with JIA was diagnosed with thyrotoxicosis or vitiligo respectively, while none was diagnosed with autoimmune thyroiditis or alopecia areata during the follow-up period. In the JIA cohort, two (0.7%) individuals were diagnosed with more than one autoimmune condition during the study period, and five (0.4%) individuals in the reference population.

**Table 2:** The occurrence of autoimmune conditions

Autoimmunity	JIA cohort (*n* = 273)	References (*n* = 1291)
All autoimmune diseases	21 (7.7%)	43 (3.3%)
Hypothyroidism	9 (3.3%)	19 (1.5%)
Coeliac disease	7 (2.6%)	9 (0.7%)
Diabetes Mellitus, type 1	5 (1.8%)	9 (0.7%)
Thyrotoxicosis	1 (0.4%)	3 (0.2%)
Vitiligo	1 (0.4%)	1 (0.1%)
Autoimmune thyroiditis	0 (0%)	7 (0.5%)
Alopecia areata	0 (0%)	1 (0.1%)
More than one autoimmune disease	2 (0.7%)	5 (0.4%)

The occurrence of the autoimmune conditions* after juvenile idiopathic arthritis (*JIA*) diagnosis in the *JIA* cohort and age- and sex matched references

^*^4 individuals lost to follow-up due to migration from the study area before their autoimmune diagnosis are included in the table since they later have re-entered the region of Skåne and have been found in the SHR

Antinuclear antibodies were present in 16 (76.2%) of the individuals with autoimmune comorbidity in the JIA group, where ANA was detected after the autoimmune diagnosis in three cases. In the JIA group without an autoimmune comorbidity, ANA were present in 134 individuals (49.1%). The number of ANA positive individuals in the reference population is unknown due to lack of information about their medical history.

### Individuals with *JIA* have increased risk of being diagnosed with autoimmune disease

**Fig. 1:**
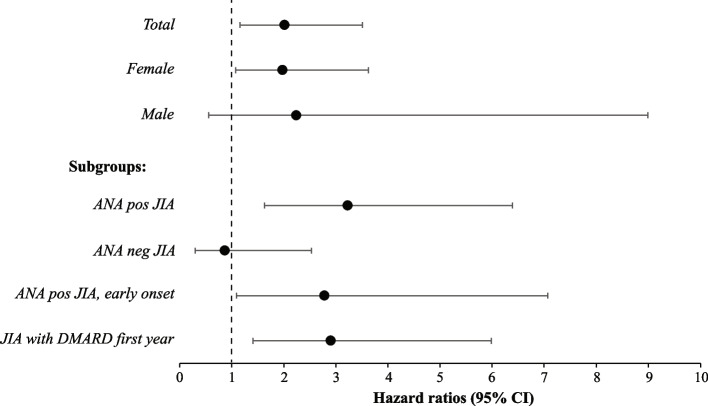
Hazard ratios for comorbid autoimmune diseases in juvenile idiopathic arthritis (JIA) compared to references. Conditional Cox proportional hazard regression models were used for the calculation of hazard ratios (HR) with 95% confidence interval (CI) for the autoimmune diseases: hypothyroidism, thyrotoxicosis, autoimmune thyroiditis, coeliac disease, type 1 diabetes mellitus, vitiligo, or alopecia areata. In all analyses, the individuals in the JIA cohort were compared to age- and sex matched references without JIA. Associations with autoimmune disease were further calculated in four subgroups, individuals belonging to; antinuclear antibodies (ANA) positive JIA, ANA negative JIA, ANA positive JIA with onset before the age of six, and JIA with treatment with any disease-modifying antirheumatic drug (DMARD) within the following calendar year after JIA diagnosis. The bars illustrate the 95% CIs with markers for HR. Number of individuals with comorbid autoimmune disease in the different analyses were in the JIA cohort; 18 in the total cohort, 15 in the female subgroup, 3 in the male subgroup, 14 in the ANA positive subgroup, 4 in the ANA negative subgroup, 7 in subgroup of the ANA positive disease with onset before the age of six, and 12 in the subgroup treated with any DMARD. Corresponding numbers were in the reference population; 42 in the total cohort, 36 in the female subgroup, 6 in the male subgroup, 20 in the reference subgroup to ANA positive disease, 22 in the reference subgroup to ANA negative disease, 12 in the reference subgroup to ANA positive disease with onset before the age of six, and 19 in the reference subgroup to treatment with any DMARD. Significant differences were found in the total JIA cohort, females, and in the subgroups; ANA positive JIA, ANA positive JIA with onset before the age of six, and JIA individuals with DMARD treatment within the following calendar year after JIA diagnosis

In total, 273 individuals with JIA and 1291 persons of the reference population were included in the conditional Cox proportional hazard regression analyses. Demographic information on the individuals analysed in the conditional Cox proportional hazard regression analyses is enclosed as Additional file 1 (see Additional file 1). Of the individuals with JIA, 208 were compared to five references, 56 to four references, and nine to three references. Hazard ratio for autoimmune disease in general was 2.01 (95% CI 1.16–3.51) (Fig. 1) for the total JIA cohort compared to the reference population. HR for autoimmune diseases was 1.98 (95% CI 1.08–3.62) for females with JIA and 2.24 (95% CI 0.56–8.99) for males with JIA. We further calculated the association with autoimmune disease in subgroups of JIA compared to the reference population. A group of individuals with JIA that needed DMARD within the following calendar year after JIA diagnosis was created as a surrogate marker for more severe disease course. Four different subgroups were then analysed; ANA positive JIA, ANA negative JIA, ANA positive with early onset (≤ 6 years) JIA, and JIA with DMARD treatment within the first year. For individuals belonging to the ANA positive JIA subgroup HR was 3.23 (95% CI 1.63–6.39). For individuals belonging to the ANA negative JIA HR was 0.87 (95% CI 0.30–2.53). Similarly, in the group with ANA positive with early onset (≤ 6 years) JIA HR was significant (HR 2.78, 95% CI 1.09–7.07). In individuals with JIA and DMARD treatment within the following calendar year after JIA diagnosis, the HR was 2.90 (95% CI 1.41–5.98).

**Fig. 2:**
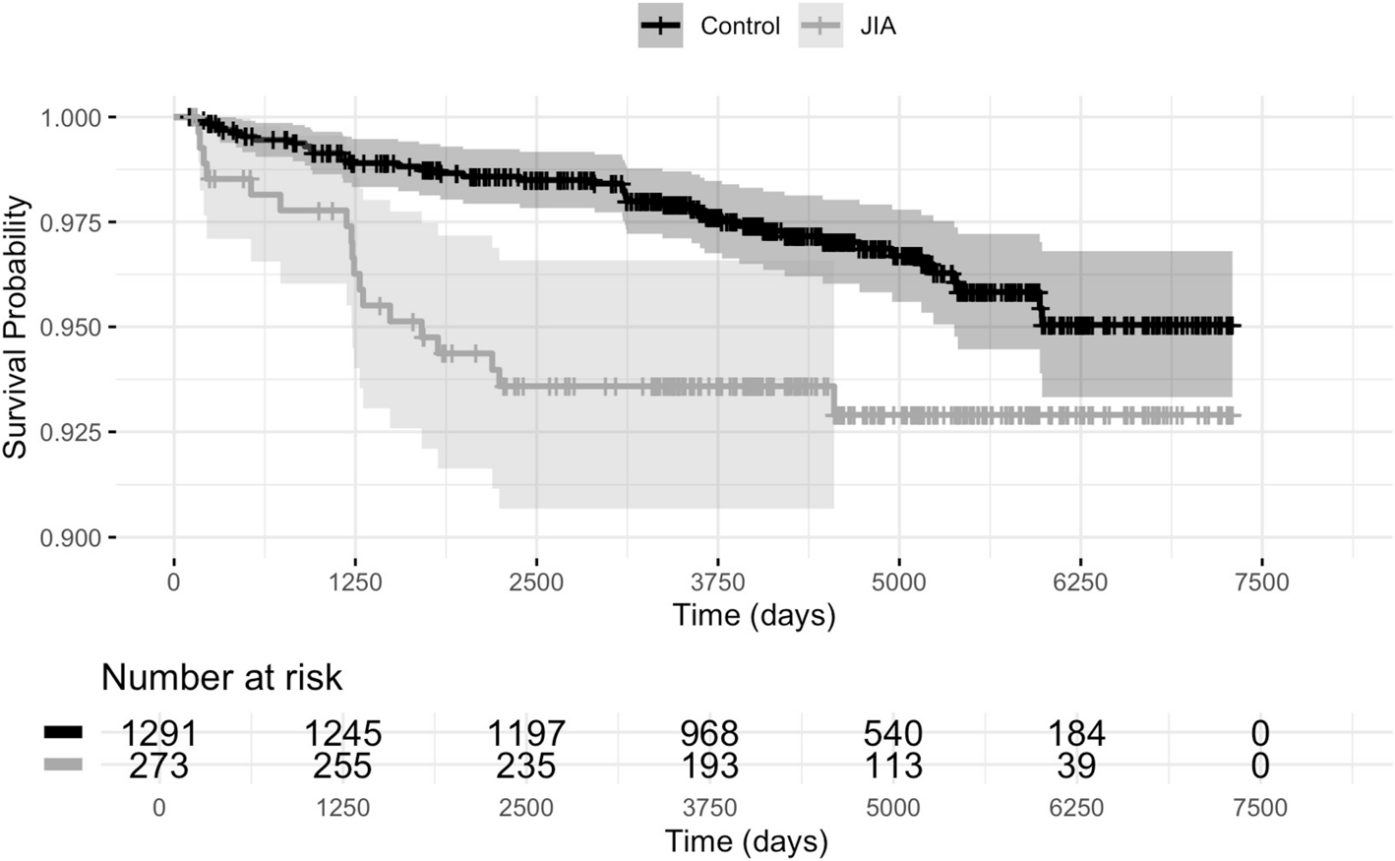
Kaplan–Meier curve for comorbid autoimmune diseases in juvenile idiopathic arthritis (JIA) compared to references. Survival curve with 95% confidence interval (CI) for the comorbid autoimmune diagnoses: hypothyroidism, thyrotoxicosis, autoimmune thyroiditis, coeliac disease, type 1 diabetes mellitus, vitiligo, or alopecia areata, in individuals with JIA and age- and sex matched references without JIA according to Kaplan–Meier analysis

Compared to the reference group, individuals with JIA had an increased risk of being diagnosed with a comorbid autoimmune disease the first years after their arthritis diagnosis (Fig. 2). When investigating the residuals of the conditional Cox proportional hazard regression analyses, there were a drift in the residuals between 2200 and 3500 days (see Additional file 2), indicating that there might be a change in risk over time. Therefore, additional analyses were limited to a 7 years’ follow-up time during which the residuals are comparable.

Results were similar when analyses were limited to the first 7 years of disease, with HR 4.11 (95% CI 2.13–7.91) for autoimmune disease compared to the references. HR for autoimmune diseases in females was 4.34 (95% CI 2.09–9.00) and 3.27 (95% CI 0.73–14.68) for males. HR was 9.29 (95% CI 3.74–23.04) for individuals belonging to the ANA positive JIA subgroup, and HR was 1.12 (95% CI 0.32–3.99) for individuals belonging to ANA negative. HR was 6.67 (95% CI 2.10–20.90) in the group with ANA positive, early onset (≤ 6 years) arthritis. HR was 7.27 (95% 2.81–18.80) in the group needing DMARD treatment within the following calendar year after JIA diagnosis.

### ANA positivity was associated with increased risk for autoimmune comorbidity within the first 7 years of disease in the *JIA* group

**Fig. 3:**
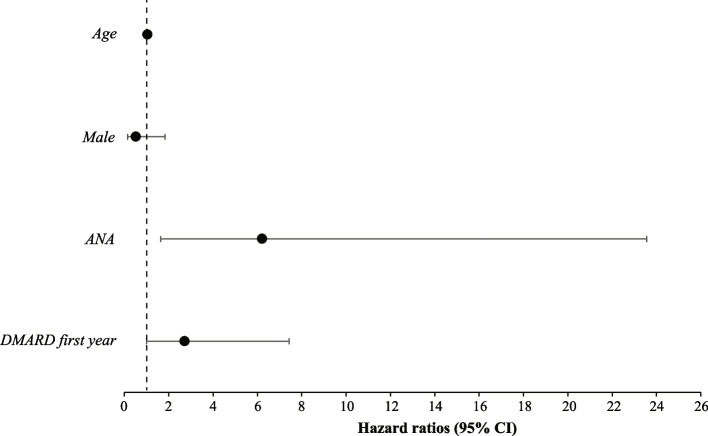
Hazard ratios for comorbid autoimmune disease in juvenile idiopathic arthritis (JIA) depending on disease characteristics. Conditional Cox proportional hazard regression models with hazard ratios (HR) and 95% confidence interval (CI) within the first 7 years after JIA diagnosis for the autoimmune conditions: hypothyroidism, thyrotoxicosis, autoimmune thyroiditis, coeliac disease, type 1 diabetes mellitus, vitiligo, or alopecia areata, stratified on the basic disease characteristics; age at JIA diagnosis, male sex, antinuclear antibodies (ANA) positivity, and treatment with disease-modifying antirheumatic drug (DMARD) within the following calendar year after JIA diagnosis. The bars illustrate the 95% CIs with markers for HR. Number of individuals with comorbid autoimmune disease in the different analyses were in the JIA cohort; 17 in the total cohort, 3 in the male subgroup, 14 in the ANA positive subgroup, and 11 in the subgroup treated with any DMARD within the following calendar year after JIA diagnosis. In individuals with JIA, ANA positivity was associated with a significantly increased risk of a comorbid autoimmune disease

We now set out to explore predictors of comorbid autoimmune diagnosis for individuals diagnosed with JIA (Fig. 3). The predictor model was tested with the patient characteristics of sex, ANA, and age, and thereafter with the characteristics of sex, ANA, age, as well as DMARD treatment within the following calendar year after JIA diagnosis. When including DMARD treatment, the model showed a better fit and was therefore included in the analyses (Akaike information criterion (181.6 vs. 183.6) and Bayesian information criterion (184.9 vs 186.1)). ANA positivity significantly increased the risk of comorbid autoimmune conditions in individuals diagnosed with JIA (HR 6.21 (95% CI 1.64–23.55)). In the group needing DMARD treatment within one calendar year after diagnosis, HR was elevated, 2.72 (95% CI 0.995–7.43), however not reaching statistical significance (*p* = 0.051). Age at JIA diagnosis and sex did not affect the risk of autoimmune comorbidity in the JIA group.

### The risk of being diagnosed with coeliac disease as well as hypothyroidism was increased within the first 7 years after *JIA* diagnosis

**Fig. 4:**
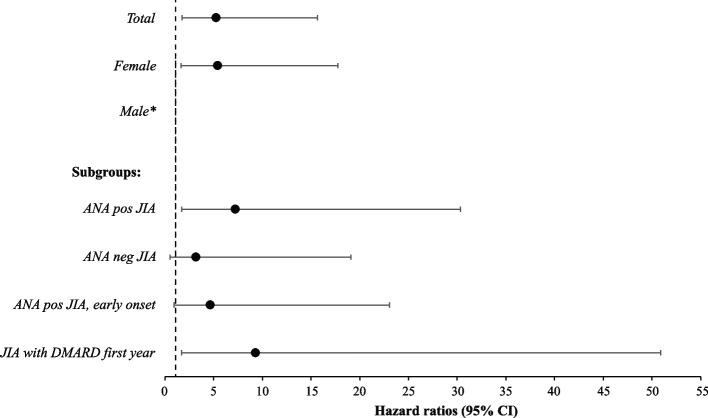
Hazard ratios for comorbid coeliac disease in juvenile idiopathic arthritis (JIA) compared to references. Conditional Cox proportional hazard regression models were used for the calculation of hazard ratios (HR) with 95% confidence interval (CI) for coeliac disease within the first 7 years after JIA diagnosis. In all analyses, the individuals in the JIA cohort were compared to age- and sex matched references without JIA. Associations with coeliac disease were calculated in the four subgroups; individuals belonging to antinuclear antibodies (ANA) positive JIA, ANA negative JIA, ANA positive JIA with onset before the age of six, and JIA with treatment with any disease-modifying antirheumatic drug (DMARD) within the following calendar year after JIA diagnosis. The bars illustrate the 95% CIs with markers for HR. Number of individuals with comorbid coeliac disease in the different analyses were in the JIA cohort; 7 in the total cohort, 6 in the female subgroup, 5 in the ANA positive subgroup, 2 in the ANA negative subgroup, 3 in the subgroup of ANA positive disease with onset before the age of six, and 4 in the subgroup treated with any DMARD. Corresponding numbers were in the reference population; 6 in the total cohort, 5 in the female subgroup, 3 in the reference subgroup to ANA positive disease, 3 in the reference subgroup to ANA negative disease, 3 in the reference subgroup to ANA positive disease with onset before the age of six, and 2 in the reference subgroup to treatment with any DMARD. Data on coeliac disease in males is not presented as there were too few events (*n* = 2) for this subgroup analysis. Significant differences were found in the total JIA population, females and in the subgroups ANA positive JIA and JIA with DMARD treatment

**Fig. 5:**
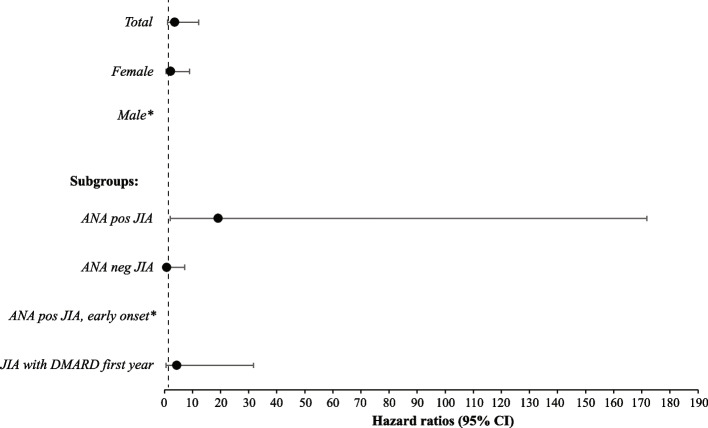
Hazard ratios for comorbid hypothyroidism in juvenile idiopathic arthritis (JIA) compared to references. Hazard ratios (HR) with 95% confidence interval (CI) for hypothyroidism within the first 7 years after JIA diagnosis using conditional Cox proportional hazard regression models. In all analyses, the individuals in the JIA cohort were compared to age- and sex matched references without JIA. Associations with hypothyroidism were calculated in the four subgroups; individuals belonging to antinuclear antibodies (ANA) positive JIA, ANA negative JIA, ANA positive JIA with onset before the age of six, and JIA with treatment with any disease-modifying antirheumatic drug (DMARD) within the following calendar year after JIA diagnosis. The bars illustrate the 95% CIs with markers for HR. Number of individuals with comorbid hypothyroidism in the different analyses were in the JIA cohort; 5 in the total cohort, 3 in the female subgroup, 4 in the ANA positive subgroup, 1 in the ANA negative subgroup, and 2 in the subgroup treated with any DMARD. Corresponding numbers were in the reference population; 6 in the total cohort, 6 in the female subgroup, 1 in the reference subgroup to ANA positive disease, 5 in the reference subgroup to ANA negative disease, and 2 in the reference subgroup to treatment with any DMARD. Data on hypothyroidism in males as well as ANA positive disease with onset before the age of six are not presented as there were too few events (*n* = 2 and 0, respectively) for these subgroup analyses. Significant differences were found in the total JIA population, and in the subgroup of individuals with ANA positive JIA

Since hypothyroidism and coeliac disease are conditions that can be asymptomatic and still be detected by blood sample screening, these conditions were chosen for separate analysis. Comparisons were now made between the entire JIA cohort and references, limited to a follow-up time of 7 years. Subgroup analyses were made, for individuals belonging to the four above mentioned JIA groups (ANA positive JIA, ANA negative JIA, ANA positive with early onset JIA, and JIA with DMARD treatment within the first year). Hazard ratio for coeliac disease was 5.24 (95% CI 1.76–15.65) in the total JIA cohort, with further increase in individuals belonging to the ANA positive subgroup (HR 7.22, 95% CI 1.72–30.35) and the subgroup treated with DMARD (HR 9.30, 95% CI 1.70–50.88) (Fig. 4). Hazard ratios for hypothyroidism was 3.74 (95% CI 1.14–12.30), with a significant elevated HR in the subgroup analysis of individuals belonging to the ANA positive JIA group (HR 19.17, 95% CI 2.14–171.80) (Fig. 5).

## Discussion

In this study we showed that individuals with JIA diagnosed in the biological era in the population-based south-Swedish JIA cohort have an increased risk of being diagnosed with a comorbid autoimmune condition compared to age- and sex-matched references from the general population. ANA positive disease was associated with a further increased risk. Our results suggest a need of laboratory screening for autoimmune conditions, particularly coeliac disease and hypothyroidism, within the first 7 years after JIA diagnosis and urges physicians to investigate symptoms indicative of comorbid autoimmune disease in individuals with JIA, especially among those with ANA positivity.

In our study, the risk of a comorbid autoimmune disease was elevated the first years after diagnosis. The shift in the risk around 7 years after JIA diagnosis does not only have clinical significance, but is also interesting when comparing to the occurrence of iridocyclitis in JIA. It is well known that the risk of iridocyclitis, or uveitis, is increased in the patient group of ANA positive disease. Furthermore, iridocyclitis usually develops the first years after JIA diagnosis, and mostly during the first 5–7 after JIA onset [1, 22]. That the risk of both comorbid autoimmune disease and iridocyclitis seems to be elevated during the same time periods raises interesting questions about the pathogenesis of JIA and warrants future studies.

Individuals with manifest JIA were diagnosed with coeliac disease more than twice as often as the age- and sex-matched reference population. The presence of coeliac disease in our cohort is in line with previously published Swedish point prevalence in JIA of 2.8% by Öman, et al. In their study, they screened JIA patients with autoantibodies against tissue transglutaminase and confirmed the diagnosis with small intestine biopsies. Two out of six cases had asymptomatic coeliac disease. Their finding of 2.8% did not support screening with antibodies against tissue transglutaminase in individuals with JIA, since their point prevalence was close to the described prevalence in the general population. The authors did however not compare the results to an age- and sex-matched reference population, which might had changed their conclusion [11]. The frequency of 2.6% in our study is lower than reported numbers from Italy [14, 16], although the risk of coeliac disease was increased among JIA cases compared to age- and sex-matched non-JIA controls from the general population [16]. The prevalence of 0.7% coeliac disease in the reference population was lower than expected, likely due to our exclusion of individuals with the studied autoimmune conditions prior to inclusion. However, the same exclusion criteria were applied to the individuals with JIA and the somewhat low prevalence among the references emphasize that they should not be considered as general population. Undiagnosed coeliac disease might lead to severe adverse effects such as malnutrition and poor growth. The result from our study shows increased risk of coeliac disease after JIA diagnosis and indicates that screening routines for coeliac disease should be considered in the clinic.

Hypothyroidism was diagnosed in 3.3% of the individuals in our JIA cohort, which was significantly more often compared to the reference population within the first 7 years after JIA diagnosis. The presence of hypothyroidism in our study was lower than in a previously published Italian study (10.1%) [14], but higher than numbers reported from Finland (0.7%) [17]. Our restriction to only include autoimmune conditions diagnosed post JIA diagnosis, contrary to the Italian study [14], may have contributed to different prevalence of hypothyroidism between the two study populations. More importantly, in the Italian study the patients were actively screened for autoimmune comorbidity with blood test, thereby possibly diagnosing also subclinical, asymptomatic patients. Hypothyroidism is also more common in the adult than paediatric population and since our cohort is diagnosed 2000–2010, only a minor portion of the individuals were in their 30’ies at the end of the study period. A longer follow-up period can potentially increase the prevalence. In line with the Pharmachild registry study exploring predictors of autoimmune thyroid disease [18], individuals with ANA had an increased risk for hypothyroidism in our study.

Our finding of increased risk of autoimmune disease in ANA positive individuals with JIA compared to ANA negative is supported by other studies showing increased presence of ANA in autoimmune thyroid disease [23, 24]. The mechanism behind the occurrence of ANA is not known, and ANA can be considered a general marker of autoimmune processes since many autoimmune diseases share common genes and pathophysiological processes. The increased risk in the ANA positive group might also reflect the paediatric distinction of this JIA subgroup compared to the other JIA subgroups with adult counterparts. This hypothesis is supported by the significantly increased risk of autoimmune comorbidities in the newly suggested JIA subgroup with ANA positive disease with early onset in our study.

Interestingly, our study further emphasises a specifically increased risk of coeliac disease in individuals with ANA positive disease. To our knowledge, the association with ANA to the risk of comorbid coeliac disease in other autoimmune diseases has been analysed in two previous studies, indicating no increased risk of coeliac disease in ANA positive JIA [25] or ANA positive autoimmune thyroid disease [23].

Our finding of elevated, however not statistically significant, risk for autoimmune conditions in the group treated with DMARD within the following calendar year after diagnosis was interesting. This group was investigated as a surrogate marker for more severe disease, and represent individuals with more inflammation possibly also affecting other organs, but can also reflect certain genetic risk factors. Previous studies exploring the risk of autoimmune conditions in individuals with DMARDs are scarce with conflicting results and some suggest lower risk of comorbid autoimmune conditions after DMARD treatment [15, 26], whereas other have found an increased risk [27]. Our study investigated those autoimmune conditions which are most relevant in the clinic and those conditions we most often see in our clinic in this age group, excluding inflammatory bowel disease and psoriasis which are included as part of the JIA diagnosis.

Our study has some limitations. Diagnosis codes for comorbid autoimmune conditions were collected from the regional healthcare register SHR with data from 1998. The SHR did not have full regional coverage of diagnosis codes registered in outpatient care, especially in primary care, until 2004 [21]. However, we believe the number of missed autoimmune cases to be few, since paediatric autoimmune conditions are primarily diagnosed in specialized healthcare facilities in Sweden. However, the lack of full coverage may have contributed to underestimation as well as overestimation of comorbid autoimmune diagnosis in JIA, depending on whether the missing diagnosis code was registered prior to or after JIA diagnosis.

Another limitation is that the autoimmune diagnoses were not confirmed by a review of the medical records or with data on prescribed insulin, levothyroxine, or gluten free nutriments. Unfortunately, this was not possible for our study. To reduce the risk of overestimation as bias, we required that an individual had a registered autoimmune diagnosis at two separate outpatient visits or at one inpatient healthcare visit. The choice of two registered codes in outpatient care has previously been investigated by a medical record review with high validation of correct diagnostics for autoimmune diseases [28].

Furthermore, we only have annual information on DMARD treatment, and therefore lack exact information of the time relation between start of DMARD treatment and diagnosis of autoimmune comorbidity, as well as objective markers of inflammation such as active joint count, and the results on the risk of DMARD treatment within the following calendar year after JIA diagnosis as a marker for more severe disease must therefore be interpreted with caution.

An alternative explanation for the higher presence of autoimmune disease in our JIA cohort compared to references is that individuals in the JIA cohort have regular healthcare visits, increasing the risk of further diagnostics. To reduce this risk, autoimmune comorbidities registered within 90 days after JIA diagnosis were excluded due to the chance of finding such conditions during arthritis diagnostics. Moreover, the references were also selected on having at least one healthcare visit during follow-up, hence not excluding individuals with other autoimmune (not included in our statistical analyses) or inflammatory conditions, where comorbidities might be detected during laboratory diagnostic at disease onset.

Further, a larger cohort would have enabled additional subgroup analysis, possibly identifying other predisposing factors for autoimmune comorbidities in JIA than ANA positive disease, that also needs to be further evaluated as potential risk factors in a larger cohort.

There are also strengths to this study. We have a well-defined study population with validated cases of JIA, including all disease severities from mild to erosive and disabling, and all states of disease activity. The cases chosen for this study have all been diagnosed in the biologic era, making the results applicable to individuals with JIA diagnosed today. Finally, our study provides longitudinal results on cumulative incidence of autoimmune comorbidities.

## Conclusions

Individuals with JIA have a significantly increased risk of acquiring a second autoimmune diagnosis, especially coeliac disease and hypothyroidism, the first 7 years after their initial JIA diagnosis. Presence of ANA is suggested as a predictor of autoimmune comorbidity. Our results emphasize awareness in physicians of additional autoimmune disorders in individuals with JIA and advocate serological screening of autoimmune conditions during the first years after JIA diagnosis. How often and for how long this screening is required needs to be further evaluated.

## Supplementary Information

Additional file 1: Title of data:“Additional file 1. Demographic information.” Description of data: A table of the clinical and serological characteristics of the 273 individuals with juvenile idiopathic arthritis (JIA) from the south-Swedish JIA cohort, diagnosed 2000-2010, and 1291 references included in the conditional Cox proportional hazard regression analyses. Disease-modifying antirheumatic drug (DMARD) first year includes both biological and conventional synthetic DMARDs prescribed within the following calendar year after JIA diagnosis.

Additional file 2: Title of data: “Additional file 2. Residuals of conditional Cox proportional hazard regression analyses of comorbid autoimmune disease.” Description of data: A figure of the residuals of the conditional Cox proportional hazard regression analyses of comorbid autoimmune disease over time in individuals with juvenile idiopathic arthritis (JIA) compared to references.

## Data Availability

The datasets generated and analysed during this current study are not publicly available as they contain information that could compromise research participant privacy. The data are available and anonymized from the corresponding author (AD) on reasonable request and appropriate permission from regulatory authorities.

## Abbreviations

ANA: Antinuclear antibodies
CI: Confidence interval
DMARD: Disease-modifying antirheumatic drug
bDMARD: Biological disease-modifying antirheumatic drug
csDMARD: Conventional synthetic disease-modifying antirheumatic drug
HR: Hazard ratio
ICD: International Statistical Classification of Diseases and Related Health Problems
ILAR: International League of Associations for Rheumatology
IQR: Interquartile range
JIA: Juvenile idiopathic arthritis
SHR: Skåne Healthcare Register
