# Supplementary material for: Screening for comorbid autoimmune disease should be considered in children with ANA positive juvenile idiopathic arthritis – results from the south-Swedish juvenile idiopathic arthritis cohort
Source: Pediatr Rheumatol Online J. 2024 Oct 19;22:92. doi: 10.1186/s12969-024-01030-x (PMC11489994; doi:10.1186/s12969-024-01030-x)
Supplement: Supplementary file 1 — Additional file 1: Title of data:“Additional file 1. Demographic information.” Description of data: A table of the clinical and serological characteristics of the 273 individuals with juvenile idiopathic arthritis (JIA) from the south-Swedish JIA cohort, diagnosed 2000-2010, and 1291 references included in the conditional Cox proportional hazard regression analyses. Disease-modifying antirheumatic drug (DMARD) first year includes both biological and conventional synthetic DMARDs prescribed within the following calendar year after JIA diagnosis. [file 12969_2024_1030_MOESM1_ESM.docx]

**Additional file 1. Demographic information**

| **Characteristics** | **JIA cohort (n=273)** | **References (n=1291)** |
| --- | --- | --- |
| Female, n (%) | 182 (66.7%) | 864 (66.9%) |
| Age at JIA diagnosis or cohort entry in years, median (IQR) | 9.6 (3.1–13.0) | 9.6 (3.0–13.1) |
| ANA positive, n (%) | 135 (49.5%) | NA |
| ANA positive early onset, n (%) | 82 (30.0%) | NA |
| DMARD first year, n (%) | 122 (46.6%) *n=262* | NA |

Clinical and serological characteristics of the 273 individuals with juvenile idiopathic arthritis (JIA) from the south-Swedish JIA cohort, diagnosed 2000-2010, and 1291 references included in the conditional Cox proportional hazard regression analyses. Disease-modifying antirheumatic drug (DMARD) first year includes both biological and conventional synthetic DMARDs prescribed within the following calendar year after JIA diagnosis.

*Abbreviations:* ANA: antinuclear antibodies, DMARD: disease-modifying antirheumatic drug, IQR: Interquartile range, NA: not applicable.
