# Supplementary figures and images for: Screening for comorbid autoimmune disease should be considered in children with ANA positive juvenile idiopathic arthritis – results from the south-Swedish juvenile idiopathic arthritis cohort
Source: Pediatr Rheumatol Online J. 2024 Oct 19;22:92. doi: 10.1186/s12969-024-01030-x (PMC11489994; doi:10.1186/s12969-024-01030-x)

Additional file 2.

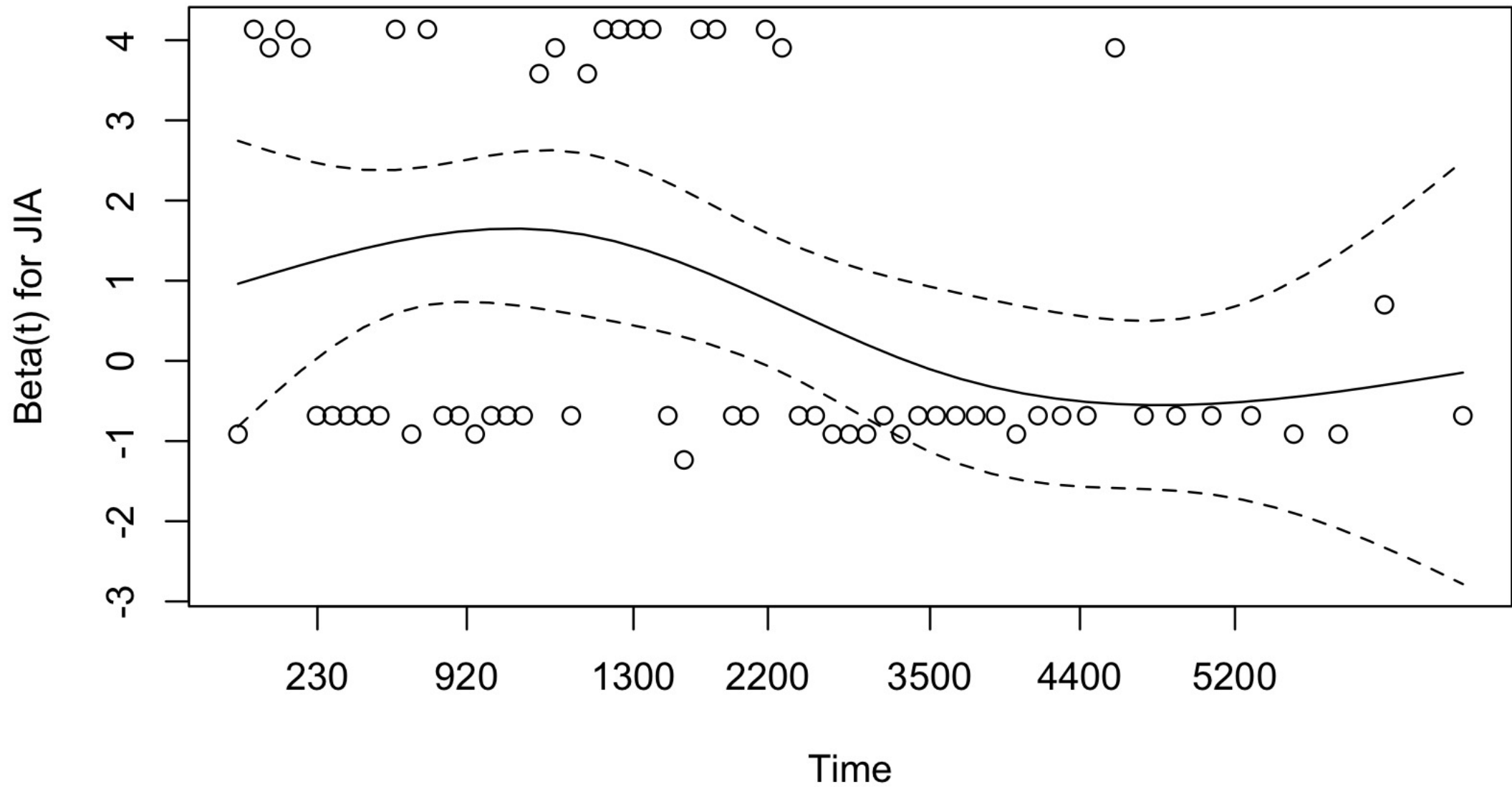

Supplement: Supplementary file 2 — Additional file 2: Title of data: “Additional file 2. Residuals of conditional Cox proportional hazard regression analyses of comorbid autoimmune disease.” Description of data: A figure of the residuals of the conditional Cox proportional hazard regression analyses of comorbid autoimmune disease over time in individuals with juvenile idiopathic arthritis (JIA) compared to references. [file 12969_2024_1030_MOESM2_ESM.pdf]
